# Cost Analysis of Integrating the PrePex Medical Device into a Voluntary Medical Male Circumcision Program in Zimbabwe

**DOI:** 10.1371/journal.pone.0082533

**Published:** 2014-05-06

**Authors:** Emmanuel Njeuhmeli, Katharine Kripke, Karin Hatzold, Jason Reed, Dianna Edgil, Juan Jaramillo, Delivette Castor, Steven Forsythe, Sinokuthemba Xaba, Owen Mugurungi

**Affiliations:** 1 United States Agency for International Development, Washington, DC, United States of America; 2 Health Policy Initiative, Futures Institute, Washington, DC, United States of America; 3 Population Services International, Harare, Zimbabwe; 4 Office of the U.S. Global AIDS Coordinator, Washington, DC, United States of America; 5 The Partnership for Supply Chain Management System, Arlington, Virginia, United States of America; 6 Zimbabwe Ministry of Health and Child Welfare, Harare, Zimbabwe; World Health Organization, Switzerland

## Abstract

**Background:**

Fourteen African countries are scaling up voluntary medical male circumcision (VMMC) for HIV prevention. Several devices that might offer alternatives to the three WHO-approved surgical VMMC procedures have been evaluated for use in adults. One such device is PrePex, which was prequalified by the WHO in May 2013. We utilized data from one of the PrePex field studies undertaken in Zimbabwe to identify cost considerations for introducing PrePex into the existing surgical circumcision program.

**Methods and Findings:**

We evaluated the cost drivers and overall unit cost of VMMC at a site providing surgical VMMC as a routine service (“routine surgery site”) and at a site that had added PrePex VMMC procedures to routine surgical VMMC as part of a research study (“mixed study site”). We examined the main cost drivers and modeled hypothetical scenarios with varying ratios of surgical to PrePex circumcisions, different levels of site utilization, and a range of device prices. The unit costs per VMMC for the routine surgery and mixed study sites were $56 and $61, respectively. The two greatest contributors to unit price at both sites were consumables and staff. In the hypothetical scenarios, the unit cost increased as site utilization decreased, as the ratio of PrePex to surgical VMMC increased, and as device price increased.

**Conclusions:**

VMMC unit costs for routine surgery and mixed study sites were similar. Low service utilization was projected to result in the greatest increases in unit price. Countries that wish to incorporate PrePex into their circumcision programs should plan to maximize staff utilization and ensure that sites function at maximum capacity to achieve the lowest unit cost. Further costing studies will be necessary once routine implementation of PrePex-based circumcision is established.

## Introduction

In 2005–2007, three randomized controlled clinical trials demonstrated that voluntary medical male circumcision (VMMC) reduced male acquisition of HIV through heterosexual intercourse by approximately 60% [1]–[3]. Since then mathematical modeling studies have suggested that scaling up VMMC in 13 Eastern and Southern African countries to 80% coverage over five years and maintaining that coverage through 2025 could avert 3.4 million HIV infections over that time period and save approximately $16.5 billion in the cost of HIV treatment and care [4].

Despite the promise of VMMC to substantially impact the HIV epidemic in these settings, scale-up of VMMC programs has been challenged by issues related to demand creation and service availability in remote and resource-constrained areas [5]–[7]. New adult VMMC devices are currently being assessed for safety, effectiveness, cost, and client and provider acceptability [8], [9]. Devices could potentially simplify the procedure, enabling non-physician providers to conduct the surgery in a wider array of settings. The availability of devices might also offer an alternative mode of VMMC for men who have fears related to conventional surgery.

One adult VMMC device, PrePex, developed by Circ MedTech (Herzelia, Israel), works by compressing the foreskin between a fitted ring and elastic band, leading to distal tissue necrosis. In most cases, PrePex does not require the use of injected anesthesia and does not require suturing. Clients who undergo VMMC using the PrePex device are required to wear the device for seven days and then return to the clinic for removal on the seventh day. In a Rwanda field study, the PrePex procedure took 4.3 minutes for device application (including preparation) and 3.8 minutes for device removal seven days later [10], compared with 23–30 minutes for conventional surgery (from scrubbing the patient in preparation for the operation to cleaning the wound after suturing) in a multi-country study [11].

A series of three studies (safety case study, comparative study, and field study) of the PrePex device was first completed in Rwanda [10], [12], [13]. This same series of studies was subsequently completed independently in Zimbabwe. The information generated from these six studies informed the WHO decision in May 2013 to add PrePex to its prequalification list [14], [15].

Claims have been advanced that the PrePex procedure would result in significantly decreased unit costs per VMMC compared with conventional surgery; this has not been borne out in analyses published to date. Using hypothetical costing information from Kenya, Obiero and colleagues derived a unit cost of $44.54–$49.02 for PrePex, not including the device cost, and $54.52–$55.29 for forceps-guided surgical VMMC [16]. Duffy and colleagues collected costs of PrePex-based and surgical VMMC at a high-volume urban hospital in Uganda and found a unit cost of $30.55 for PrePex-based and $22.65 for surgical VMMC, using a PrePex device price of $20 and excluding demand creation costs [17].

Countries in Eastern and Southern Africa have already begun scaling up VMMC using conventional surgical approaches. Because of age and other exclusions (PrePex is only qualified for use with men over the age of 18), programs that wish to introduce PrePex will need to continue to make conventional surgery available. Therefore, we sought to examine potential cost implications of integrating the PrePex device into an existing VMMC program. Instead of estimating a unit cost specifically for PrePex-based VMMC vs. surgical VMMC, as others have [16], [17], we examined the site-level unit cost of VMMC at a high-throughput public facility providing only routine surgical VMMC (“routine surgery site”) and at a similar facility in which staff and equipment were added to also conduct PrePex-based circumcisions for a research study (“mixed study site”). We used data collected during routine surgical implementation in Zimbabwe and as part of the Zimbabwe PrePex field study, respectively. Because of the differing staffing pattern and equipment used at each site, the unit costs of the two sites are not comparable. Therefore, to enable comparison of the unit costs, we also created a hypothetical mixed site scenario that retained the same staffing pattern and number of beds as the routine surgery site.

This study poses several questions: (1) What are the major drivers of unit costs in each type of site (routine surgery and the two different mixed sites)? (2) How would the unit cost at a mixed site change with varying ratios of surgical to PrePex-based circumcisions? (3) How would unit costs change with varying levels of site utilization? (4) What impact would different device prices have on the unit cost?

## Methods

### Implementation models

**Figure 1:**
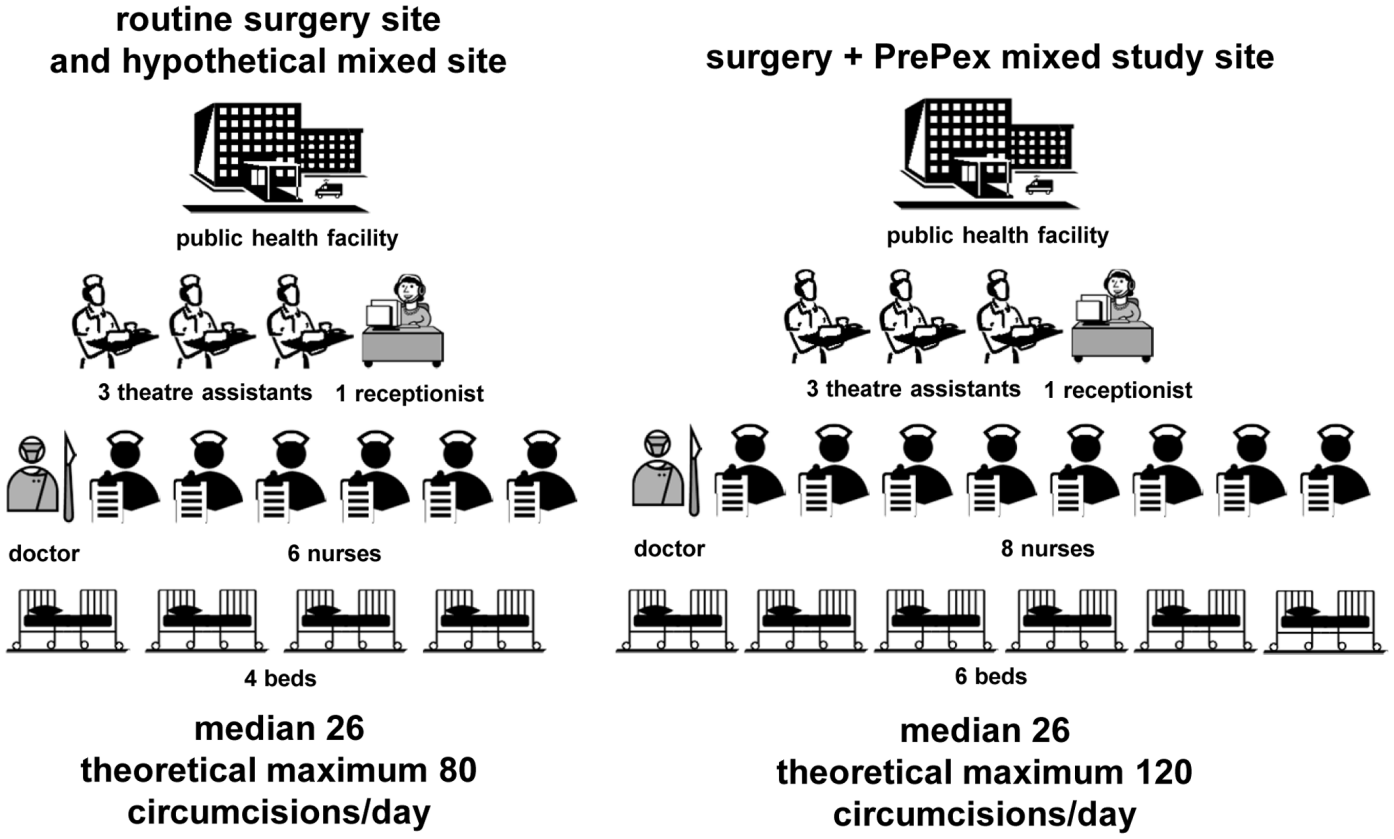
Key characteristics of the different implementation models. The routine surgery site and the hypothetical mixed site employed 1 medical doctor, 6 nurses, 3 theatre assistants, and 1 receptionist in a 4-bed facility, and the mixed study site employed 1 medical doctor, 8 nurses, 3 theatre assistants, and 1 receptionist in a 6-bed facility.

Three implementation models were evaluated (Figure 1): a routine surgery site based on the configuration (staffing, equipment) of the Bulawayo VMMC Centre, a mixed study site based on the configuration of the Harare PrePex Field Study Site, and a hypothetical mixed site with a configuration comparable to that of the routine surgery site. The Bulawayo and Harare sites are dedicated VMMC sites, where efficiencies such as task sharing, use of VMMC kits with disposable surgical instruments for the conventional surgery, and use of multiple surgical beds for each surgeon are being implemented.

The routine surgery site had a four-bed capacity and employed one medical doctor, six nurses, three theatre assistants, and one receptionist. The doctors had overall responsibility for the surgical procedure, provided local anesthesia, removed the foreskin, stopped bleeding either using sutures or electrocauterization, and attended to post-operative complications and treatment of adverse events involving wound revisions or severe infections with abscesses. The nurses conducted group education sessions, conducted pre-operative examination and counseling, conducted HIV testing and counseling, assisted with circumcisions, conducted post-operative examination and counseling, and conducted post-operative review of the client on day 2,day 7 and day 42 post-surgery. The theatre assistant was responsible for cleaning the operation room, cleaning instruments after the operation, preparing instruments and operation room in case of adverse events, and cleaning the waiting area, counseling and examination rooms. The receptionist was responsible for taking clients' personal details, explaining the procedure, and booking the client for operation and follow-up review.

The mixed study site had a six-bed capacity and employed one medical doctor, eight nurses (an additional two nurses and two beds to conduct PrePex circumcisions), three theatre assistants, and one receptionist. In addition to the responsibilities outlined above for the surgery site, the doctor supervised the nurses conducting PrePex circumcisions, handled device-related complications, and conducted surgery in case of displacement of the device. The nurses conducted the PrePex procedure: fitting the PrePex device size, placing the device, removing the dried foreskin, inner ring, and elastic band on day 7, dressing the wound, and conducting post-operative review of the client after removal of the device on day 7 and day 42. The theatre assistant sterilized instruments used for removal of the foreskin and device. All staff at the mixed site were qualified to conduct both types of circumcisions based on client demand.

The hypothetical mixed site had a four-bed capacity and employed the same staff as the routine surgery site.

Data on service delivery were collected between May 8 and July 9, 2012, at the Harare site for the mixed study site model and at the Bulawayo site for the routine surgery site model. For PrePex-based circumcisions, clients who presented for placements were counted as PrePex VMMCs conducted. All devices were removed at day 7. The median number of daily circumcisions per site during this period was used in all of the unit cost calculations except the analysis that examined different levels of site capacity utilization, which used the observed minimum, first quartile, median, third quartile, maximum, and theoretical maximum (explained in the Results section) numbers of clients per day. The population to be circumcised included males ages 10 to 49 years; those under 18 years of age, those declining to participate in the study, and those with exclusions such as HIV infection, cracked foreskin, phimosis, short or tight frenulum, or preputial adhesions were not eligible to be circumcised using PrePex and were offered conventional surgery. Those with active genital infections were treated and asked to come back for circumcision at a later date. In the mixed study sites during the period of data collection, 84% of the circumcisions were conducted using conventional surgery and 16% were conducted using PrePex. This ratio was applied as described below when calculating the unit cost at the mixed study site.

### Cost Model

All analyses were performed using Microsoft Excel 2010 (Microsoft Corporation, Redmond, WA). All costs are presented in USD.

Unit costs per circumcision (regardless of site type) comprised seven cost categories: consumables, device, supply chain management, staff, durable equipment, training, and waste management. Indirect costs, costs for demand creation, and costs for complications were not included in this analysis. Unit costs represent costs to the service provider and do not include costs to clients, such as transport to and from the intervention sites. The VMMC unit cost for the mixed sites is the average unit cost of all circumcisions provided, including both conventional surgical VMMCs and PrePex-based VMMCs; we did not disaggregate unit costs by modality. Site-level unit costs were derived by calculating the per circumcision cost for each of the seven cost categories and then adding the costs of all the categories:
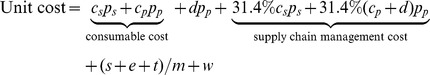
where ***c_s_*** is the per circumcision cost of consumables for conventional surgical circumcisions

***p_s_*** is the percentage of conventional surgical circumcisions at the site (for the routine surgery site, ***p_s_*** = 100%)

***c_p_*** is the per circumcision cost of consumables for PrePex-based circumcisions

***p_p_*** is the percentage of PrePex-based circumcisions at the site (for the routine surgery site, ***p_p_*** = zero)

***d*** is the device price

31.4% of the cost of consumables (including device) constitutes the supply chain cost [18]

***s*** is the annual staff cost at that type of site

***e*** is the annual durable equipment cost at that type of site

***t*** is the annual training cost at that type of site

***m*** is the median number of circumcisions performed per day at that type of site, multiplied by 220 working days per year

***w*** is the cost of waste management per circumcision

Details about each cost category follow.

#### Consumables

Lists of required consumables for both surgical VMMC using the forceps-guided technique and PrePex-based circumcisions were provided by the Zimbabwe PrePex Field Study team, as defined in the study protocol. Consumables prices were provided by the PEPFAR funded through USAID Partnership for Supply Chain Management System (SCMS) project and are available in Table S1 and Table S2. For conventional surgical VMMC, the study utilized pre-sterilized, disposable commodities bundled into kits (market price sourced by SCMS as of February 15, 2013), while a combination of disposable and reusable commodities was used for the PrePex procedure. The commodity costs for the PrePex procedure were derived from average SCMS pricing from procurements in 2009–2012, with data compiled on January 10, 2013, or from a quotation obtained from Circ MedTech on December 19, 2012, as indicated in Table S2. The actual PrePex device is disposable/single-use. Each reusable commodity cost was divided across 150 procedures to derive the unit cost, based upon experience in the field study.

#### Device

The baseline PrePex device cost applied was USD $20 per unit based on a quotation from Circ MedTech to SCMS on December 19, 2012. This is not the price that was used in the series of clinical studies in Zimbabwe, but it was the price quoted by the manufacturer for pilot implementation studies in several countries.

#### Supply chain management

Based on a study of the existing supply chain management system in Zimbabwe conducted by the USAID DELIVER Project in 2010, supply chain costs per circumcision were calculated by multiplying the costs of consumables (including device) by 31.4%, comprising 11.4% for procurement costs plus 20% for logistics expenses [18].

#### Staff salaries

Current (2012) salaries of public-sector staff involved in the VMMC program in Zimbabwe were used: medical doctor, $2,200 per month; nurse, $700 per month; theatre assistant, $150 per month; receptionist, $250 per month. These salary costs are uniform for all sites where public-sector cadres are used. Monthly salaries were multiplied by twelve to produce an annual salary and then divided by the number of circumcisions per year to produce per circumcision staff costs.

#### Durable equipment

The list of durable equipment utilized by each type of site was provided by the Zimbabwe PrePex Field Study team. Costs for each item were derived from average SCMS pricing from procurements in 2009–2012, with data compiled on January 10, 2013, and listed in Table S3. Equipment costs were allocated uniformly over three years based on estimated life span.

#### Training

All nurses and doctors in the routine surgery and mixed sites received the same initial competency-based team training. Staff turnover was approximately 50% per year, so on average an entirely new cohort of staff would need to be retrained every 3.6 years. Therefore, training costs were allocated uniformly over 3.6 years.

At the routine surgery site, the training cost in 2012 was USD $884 per trainee for a 6-day course that included 2.5 days of theory and 3.5 days of applied work. For the mixed sites, the training cost was USD $1,252 per trainee for an 8.5-day course that included an additional 2.5 days for applied training with PrePex. All nurses and doctors at the mixed site were trained in both surgical and PrePex-based circumcision.

#### Waste management

At the routine surgery site, according to 2012 site records, the cost of waste management of USD $1.00 per kg of waste was based on current program generation of 25 kg of waste per week, resulting from 130 circumcisions per week. The same unit cost for waste management per VMMC was applied to the mixed sites.

## Results

### Key cost drivers

The unit cost per VMMC for the routine surgery site was $55.83, for the mixed study site it was $60.58, and for the hypothetical mixed site it was $57.45 (Table 1). The two largest contributors to the unit cost were consumables and staff. At the routine surgery site, consumables ($30.36) and staff ($14.90) contributed a combined 81% to the unit cost; in the mixed study site, consumables ($30.87), including device, and staff ($17.83) contributed a combined 80% to the unit cost; in the hypothetical mixed site, consumables ($30.87), including device, and staff ($14.90) contributed a combined 80% to the unit cost. Training, durable equipment, and waste management contributed negligibly to the unit costs at all three sites.

**Table 1:** Cost drivers for unit cost of different implementation models.

	Routine surgery site		Mixed study site*		Hypothetical mixed site*	
Cost category	cost per circumcision	% of unit cost	Cost per circumcision	% of unit cost	Cost per circumcision	% of unit cost
Staff	$14.90	27%	$17.83	29%	$14.90	26%
Training	$0.30	0.5%	$0.58	1.0%	$0.45	0.8%
Consumables	$30.36	54%	$27.62	46%	$27.62	48%
Device	$0.00	0%	$3.25	5%	$3.25	6%
Durable equipment	$0.55	1.0%	$1.42	2.3%	$1.35	2.4%
Supply chain management	$9.53	17%	$9.69	16%	$9.69	17%
Waste management	$0.19	0.3%	$0.19	0.3%	$0.19	0.3%
**Total unit cost/circumcision**	**$55.83**		**$60.58**		**$57.45**	

*84% surgery+16% PrePex.

### Impact of ratio of conventional surgical to PrePex-based circumcisions at mixed site

At the mixed study site between May 8 and July 9, 2012, 28% of the clients were ineligible for PrePex due to being less than 18 years old, and 5.6% were ineligible for physiological reasons such as phimosis or tight foreskin. Since acceptability of the PrePex device in Zimbabwe outside the study environment is currently unknown, we looked at the effect on the unit cost of varying the ratio of conventional surgical circumcisions to PrePex-based circumcisions. The maximum percentage of PrePex-based circumcisions with 100% acceptability would be 68%, given age and physiological exclusions. We kept the staffing and the total number of circumcisions per day constant in this analysis: although in theory both the staffing and the total number of circumcisions per day might change with different ratios of the different types of circumcisions, we did not have robust data upon which to base changes in these variables for this analysis. The unit cost in this analysis ranged from $60 per circumcision when 100% of circumcisions were performed using conventional surgery to $63 per circumcision when 68% of circumcisions were performed using PrePex (Table 2).

**Table 2:** Effect of proportion of conventional surgical versus PrePex-based circumcisions on unit cost at the mixed study site.

% conventional surgical circumcisions	% PrePex-based circumcisions	Unit cost
100%	0%	$60*
95%	5%	$60
90%	10%	$60
80%	20%	$61
70%	30%	$61
60%	40%	$62
50%	50%	$62
40%	60%	$62
32%	68%	$63

* This number is different from the unit cost at the routine surgery site because additional equipment and staff were added at the mixed study site, but there was no increase in the number of circumcisions conducted per day.

### Utilization of site capacity

Generating, maintaining, and predicting fluctuations in demand for VMMC over time can be challenging. In some countries scaling up VMMC, sites have been fully staffed and equipped, only to experience suboptimal utilization during some periods. We examined the impact of different levels of site utilization on the unit cost. In these analyses the daily costs for staffing, durable equipment, and training were held constant, while consumables, device, supply chain management, and waste management costs varied by number of circumcisions. The distribution of numbers of circumcisions per day was based on site utilization data from the routine surgery and mixed study sites between May 8 and July 9, 2012. Because the sites were not operating at full capacity, a theoretical maximum number of circumcisions per day was estimated based on the complement of staff and equipment at each type of site in an eight-hour day if demand for circumcisions met or exceeded supply. The median number of VMMC procedures per day was 26 at both the routine surgery site and the mixed study site. The theoretical maximum number of daily VMMCs was 80 at the routine surgery site and 120 at the mixed study site. VMMC unit cost was highly sensitive to the level of site utilization in both types of sites, ranging at the mixed study site from $45 at the theoretical maximum utilization to $98 when the site performed only nine VMMCs per day (the minimum observed during the two-month period at this site). Similarly, at the routine surgery site, the unit cost ranged from $45 at theoretical maximum site utilization to $122 when only five VMMCs (the minimum observed during the two-month period at this site) were performed per day (Table 3).

**Table 3:** Effect of site capacity utilization on unit cost at routine surgery site and mixed study site.

	Routine surgery site^1^		Mixed study site^2^	
	# circ/day	Unit cost	# circ/day	Unit cost
Minimum	5	$122	9	$98
First quartile	15	$67	17	$71
Median	26	$56	26	$61
Third quartile	31	$53	33	$56
Actual maximum	56	$47	58	$50
Theoretical maximum	80	$45	120	$45

1 4 beds, 7 medical staff.

2 6 beds, 9 medical staff.

### Impact of device price

The price of the PrePex device outside of small procurements for research studies has not been negotiated. Because only 16% of circumcisions used PrePex at the mixed sites, we modeled the variations in unit cost as a function of a variety of PrePex device prices using a fictional scenario in which 68% of the circumcisions were performed using PrePex (the maximum possible in this population given age and physiological exclusions), in order to see the maximum possible impact of variations in device price. Under these assumptions, the unit cost ranged from $50 at a device price of $2.00 (device 3% of the unit cost) to $63 at a device price of $20 (device 22% of unit cost) (Table 4). Therefore, the unit cost is sensitive to variations in the device price.

**Table 4:** Sensitivity analysis of device price on unit cost at mixed study site with 68% of circumcisions conducted using the PrePex device.

Device price	Unit cost	Device % of unit cost
$2	$50	3%
$5	$52	6%
$10	$56	12%
$15	$59	17%
$20	$63	22%

## Discussion

In this analysis we sought to examine the impact on VMMC unit costs of introducing PrePex into an existing routine surgical VMMC program in Zimbabwe. Introduction of PrePex at the study site did not have a large impact on the overall unit cost. The key cost drivers for both the routine surgery and the mixed sites were consumables and staff salary costs, suggesting areas of focus for lowering the price per VMMC. At the mixed study site, the unit cost only increased by $3 when the ratio of surgical and PrePex-based circumcisions was hypothetically varied from 100% surgery to 32% surgery and 68% PrePex. Unit costs were highly sensitive to potential variations in the device price when 68% of the circumcisions were performed using the device; therefore, a responsibly low public-sector price could result in cost savings and avail VMMC services to more people in need, if acceptability of PrePex turns out to be high.

The largest impact on the unit cost was underutilization of site capacity. At the mixed study site, the minimum observed daily service utilization resulted in unit costs twice those of maximum observed service utilization. Similar cost increases were seen from underutilization at the routine surgery sites. The negative impact of underutilization would be even greater if overhead costs had been included in the analysis. This result highlights the importance of optimal demand creation and a balanced relationship between service supply (availability) and demand for VMMC services, regardless of the circumcision method used.

Further research examining alternative service delivery models in different settings and country contexts might find unit costs that are different from those presented in this study. For example, if implementation is carried out in a site dedicated entirely to VMMC, rather than co-located or integrated within a public health care facility, increased costs for waste transportation and disposal and supply chain management might be expected with either the routine surgery or mixed site model. If circumcisions are outsourced to the private sector, staff salaries, facility rental or construction, and profit may increase or decrease the unit costs. The use of temporary or locum staff to adjust capacity to accommodate fluctuations in demand could change the cost structure. Different implementation models—for example, those involving mobile teams and outreach campaigns—might include additional or higher costs for expenses such as transportation. If a new supply chain management system needs to be created or the existing one strengthened, supply chain management costs would likely be higher, at least temporarily. If commodity transportation requires air freight rather than ocean freight, procurement costs will be higher. The teams in Zimbabwe were highly experienced and efficient, so they could conduct many circumcisions per day. Newly trained teams usually need more time to conduct each circumcision, reducing productivity and driving up unit costs.

It is also reasonable to speculate that unit costs could decrease as a result of PrePex availability. At the point that they are no longer experimental, PrePex circumcisions provided as a routine service could be reduced in cost due to potential reductions in consumables and supply chain costs and higher service utilization. A responsible public-sector price for the PrePex device when purchased in large volumes, which reflects the costs of materials and production plus a reasonable mark-up for profit, should reduce the overall consumables cost. Because supply chain costs are related to consumables costs, by extension they might also decrease.

In Zimbabwe, nurses are not allowed to perform certain aspects of the surgical male circumcision procedure, but the PrePex Field Study demonstrated that nurses are able to conduct the entire PrePex VMMC procedure. Different staffing patterns can affect the overall unit cost. For countries without task shifting, instituting a task-shifting policy is one way to significantly decrease staff costs and therefore decrease unit costs of VMMC.

This study has several limitations. The per-VMMC unit costs provided did not include all cost components, and they were based on limited data using a single service delivery model. PrePex procedures were performed as part of a study, and thus the costs of those procedures do not reflect routine service delivery conditions (for example, in Zimbabwe the mixed sites employed more staff than those sites offering routine circumcision, but did not observe an increase in the total number of circumcisions. In routine service delivery, it is doubtful that such additional staff would be hired without any increase in uptake). Conventional surgical circumcisions, on the other hand, were provided as a routine service, in a model perfected over years. Actual unit costs should be determined by careful costing studies in a large number of sites once routine implementation of both circumcision methods is well under way. The unit costs did not include indirect costs or costs for demand creation, each of which might contribute significantly to the unit cost. Other groups have attempted to derive unit costs for demand creation for VMMC in other settings, but they have found too much variation in implementation and costs to be able to produce a standard unit cost [19]. Because of the importance of demand creation, this question merits further research. At this time, little is known about the acceptability of the PrePex device outside of research settings, either in Zimbabwe or in other cultural contexts. Costs of supervision, community engagement, and program management were not included in this analysis. These costs should be collected as the introduction of PrePex is rolled out on a larger scale in a number of different settings.

This study provides new data on the cost of introducing the PrePex device into an existing surgery-based VMMC program and highlights the importance of controlling consumable and staff costs and ensuring high levels of site utilization through effective demand creation strategies. We found no evidence in these analyses that introducing the PrePex device would result in increased efficiency of the VMMC program in terms of reducing the unit cost. To assist countries that wish to incorporate PrePex into their VMMC scale-up plans, it will be necessary to collect a broader diversity of actual cost data from different implementation models, across different countries.

## Supporting Information

Table S1 **Itemized consumables costs for forceps-guided routine surgical circumcisions.**
(DOCX)

Table S2 **Itemized consumables costs for PrePex-based circumcisions.**
(DOCX)

Table S3 **Durable equipment costs for the routine surgery site, mixed study site, and hypothetical mixed site.**
(DOCX)
