# Supplementary material for: Cost Analysis of Integrating the PrePex Medical Device into a Voluntary Medical Male Circumcision Program in Zimbabwe
Source: PLoS One. 2014 May 6;9(5):e82533. doi: 10.1371/journal.pone.0082533 (PMC4011574; doi:10.1371/journal.pone.0082533)
Supplement: Table S1 — Itemized consumables costs for forceps-guided routine surgical circumcisions. (DOCX) [file pone.0082533.s001.docx]

# SUPPLEMENTAL DIGITAL CONTENT

Table S1: Itemized consumables costs for forceps-guided routine surgical circumcisions

| **Product** | **Pack cost** | **Packs per procedure** | **Cost per procedure** |
| --- | --- | --- | --- |
| MC disposable instrument kit | $14.01 | 1 | $14.01 |
| Surgical gloves (pair) | $0.34 | 4 | $1.36 |
| Diathermy cautery tips (single) | $2.77 | 1 | $2.77 |
| Lidocaine HCL 1% (20 ml vial) | $0.47 | 1 | $0.47 |
| Bupivacaine HCL 0.5% (20 ml) | $2.68 | 0.2 | $0.54 |
| Paracetamol 500 mg (1,000 tabs) | $10.00 | 0.02 | $0.20 |
| Povidone iodine 10% solution (200 ml bottle) | $1.03 | 0.3 | $0.31 |
| Compression bandage, self-adherent (162 meters) | $22.32 | 0.005 | $0.10 |
| Suture, braided, absorbable (12 pieces) | $3.60 | 0.01 | $0.04 |
| Infection prevention supplies | $6.66 | 1 | $6.66 |
| Emergency commodities | $0.66 | 1 | $0.66 |
| STI testing and treatment commodities | $3.25 | 1 | $3.25 |
| **TOTAL** | | | **$30.36** |
