# Supplementary material for: Cost Analysis of Integrating the PrePex Medical Device into a Voluntary Medical Male Circumcision Program in Zimbabwe
Source: PLoS One. 2014 May 6;9(5):e82533. doi: 10.1371/journal.pone.0082533 (PMC4011574; doi:10.1371/journal.pone.0082533)
Supplement: Table S2 — Itemized consumables costs for PrePex-based circumcisions. (DOCX) [file pone.0082533.s002.docx]

Table S2: Itemized consumables costs for PrePex^TM^-based circumcisions

| **Product** | **Price source** | **Pack cost** | **Packs per procedure** | **Cost per procedure** | **Notes** |
| --- | --- | --- | --- | --- | --- |
| **Placement kit** |  |  |  |  |  |
| Exam latex, non-sterile (100 pieces) | SCMS | $6.00 | 0.04 | $0.24 | Assumed 4 pieces per patient |
| Antiseptic solution, 2% clorhexidine (1,000 ml) | SCMS | $3.17 | 0.02 | $0.06 | Assumed 20 ml per patient |
| Sterile gauze pads (100 pieces) | SCMS | $6.90 | 0.03 | $0.21 | Assumed 3 swabs per patient |
| Sizing plate | CircMedTech | $0.01 | 1 | $0.01 | Produced by Circ MedTech, so price is estimated |
| Skin marker (set of 24) | SCMS | $14.00 | 0.0008 | $0.01 | Assumed 50 uses per marker |
| Anesthetic dermal cream, 5% (20 grams) | SCMS | $3.50 | 0.05 | $0.18 | Assumed 1 gram per patient as per manufacturer |
| Nurse utility scissors | CircMedTech | $1.89 | 0.007 | $0.01 | Assumed 150 uses |
| PrePex^TM^ device | CircMedTech | $20.00 | 1 | $20.00 | Produced by Circ MedTech, so price is estimated |
| **Removal kit** |  |  |  |  |  |
| Pain killer (paracetamol tabs of 500 mg; 1,000 tabs) | SCMS | $10.00 | 0.02 | $0.20 | Assumed 20 tabs per circumcision |
| Exam latex, non-sterile (100 pieces) | SCMS | $6.00 | 0.04 | $0.26 | Assumed 4 per patient |
| Antiseptic solution, 2% clorhexidine (1,000 ml) | SCMS | $3.17 | 0.02 | $0.06 | Assumed 20 ml per patient |
| Sterile gauze pads (100 pieces) | SCMS | $6.90 | 0.04 | $0.28 | Assumed 4 swabs per patient |
| Mosquito clamp straight | CircMedTech | $2.02 | 0.007 | $0.01 | Assumed 150 uses |
| Harvey wire scissors | CircMedTech | $1.93 | 0.007 | $0.01 | Assumed 150 uses |
| Spatula | CircMedTech | $1.79 | 0.007 | $0.01 | Assumed 150 uses |
| Scalpel blade (box of 20) | SCMS | $9.64 | 0.05 | $0.48 | Disposable |
| Autoclave wrap per unit | SCMS | $0.14 | 1 | $0.14 | Assumed 1 needed for autoclaving of instruments |
| Chemical indicators per unit | SCMS | $0.16 | 1 | $0.16 | Assumed 1 needed for verification of autoclaving |
| Povidone iodine, 10% solution (200 ml bottle) | SCMS | $1.03 | 0.025 | $0.03 | Assumed 5 ml per patient |
| Wound dressing; coated fabric tape with no pad | SCMS | $0.44 | 1 | $0.44 | No product found commercially; requires mixing of products and own sterility package; value based on SCMS prior experience with similar products |
| Paper tape (36 meters) | SCMS | $16.63 | 0.006 | $0.10 | Assumed 22.5 cm |
| Infection prevention supplies | SCMS | $6.66 | 1 | $6.66 | Same as surgery |
| Emergency commodities | SCMS | $0.66 | 1 | $0.66 | Same as surgery |
| STI testing and treatment commodities | SCMS | $3.25 | 1 | $3.25 | Same as surgery |
| **TOTAL** | | | | **$33.47** | |
