# Supplementary material for: Cost Analysis of Integrating the PrePex Medical Device into a Voluntary Medical Male Circumcision Program in Zimbabwe
Source: PLoS One. 2014 May 6;9(5):e82533. doi: 10.1371/journal.pone.0082533 (PMC4011574; doi:10.1371/journal.pone.0082533)
Supplement: Table S3 — Durable equipment costs for the routine surgery site, mixed study site, and hypothetical mixed site. (DOCX) [file pone.0082533.s003.docx]

Table S3: Durable equipment costs for the routine surgery site, mixed study site, and hypothetical mixed site

| **Product** | **Quantity** | | | **Cost** | | |
| --- | --- | --- | --- | --- | --- | --- |
|  | Routine surgery site^1^ | Mixed study site^2^ | Hypothetical mixed site^3^ | Routine surgery site | Mixed study site | Hypothetical mixed site |
| Examining bed | 4 | 6 | 4 | $523.60 | $785.40 | $523.60 |
| Operating stool | 8 | 12 | 8 | $380.00 | $570.00 | $380.00 |
| Standing lamp | 4 | 6 | 4 | $336.00 | $504.00 | $336.00 |
| Step ladder | 4 | 6 | 4 | $104.72 | $157.08 | $104.72 |
| IV stand | 4 | 6 | 4 | $248.00 | $372.00 | $248.00 |
| Wheelchair | 1 | 1 | 1 | $100.10 | $100.10 | $100.10 |
| Recovery bed | 2 | 2 | 2 | $920.00 | $920.00 | $920.00 |
| Recovery chair | 10 | 10 | 10 | $525.00 | $525.00 | $525.00 |
| Patient trolley | 1 | 1 | 1 | $408.10 | $408.10 | $408.10 |
| Instrument trolley | 4 | 6 | 4 | $657.56 | $986.34 | $657.56 |
| Diathermy machine | 4 | 4 | 4 | $3,600.00 | $3,600.00 | $3,600.00 |
| Diathermy pencil | 8 | 8 | 8 | $40.00 | $40.00 | $40.00 |
| Diathermy plate and cables | 8 | 8 | 8 | $360.00 | $360.00 | $360.00 |
| Defibrillator | 1 | 1 | 1 | $1,200.00 | $1,200.00 | $1,200.00 |
| Autoclave | 0 | 1 | 1 | - | $13,792.00 | $13,792.00 |
| **TOTAL** | | | | **$9,403.08** | **$24,320.02** | **$23,195.08** |

^1^ 4-bed site

^2^ 6-bed site

^3^ 4-bed site
